# High-Intensity Focused Ultrasound Induces Adipogenesis via Control of Cilia in Adipose-Derived Stem Cells in Subcutaneous Adipose Tissue

**DOI:** 10.3390/ijms23168866

**Published:** 2022-08-09

**Authors:** Seyeon Oh, Hyoung Moon Kim, Sosorburam Batsukh, Hye Jin Sun, Taehui Kim, Donghwan Kang, Kuk Hui Son, Kyunghee Byun

**Affiliations:** 1 Functional Cellular Networks Laboratory, Lee Gil Ya Cancer and Diabetes Institute, Gachon University of Medicine, Incheon 21999, Korea; 2 Department of Anatomy & Cell Biology, Gachon University College of Medicine, Incheon 21936, Korea; 3 Jeisys Medical Inc., Seoul 08501, Korea; 4 Department of Thoracic and Cardiovascular Surgery, Gachon University Gil Medical Center, Gachon University, Incheon 21565, Korea

**Keywords:** high-intensity focused ultrasound, adipogenesis, adipose-derived stem cells, cilia, skin rejuvenation

## Abstract

During skin aging, the volume of subcutaneous adipose tissue (sWAT) and the adipogenesis potential of adipose-derived stem cells (ASCs) decrease. It is known that the shortening of cilia length by pro-inflammatory cytokines is related to the decreased adipogenic differentiation of ASCs via increase in Wnt5a/β-catenin. High-intensity focused ultrasound (HIFU) is known to upregulate heat shock proteins (HSP), which decrease levels of pro-inflammatory cytokines. In this study, we evaluated whether HIFU modulates the cilia of ASCs by upregulating HSP70 and decreasing inflammatory cytokines. HIFU was applied at 0.2 J to rat skin, which was harvested at 1, 3, 7, and 28 days. All results for HIFU-applied animals were compared with control animals that were not treated. HIFU increased expression of HSP70 and decreased expression of NF-κB, IL-6, and TNF-α in sWAT. HIFU decreased the expression of cilia disassembly-related factors (AurA and HDAC9) in ASCs. Furthermore, HIFU increased the expression of cilia assembly-related factors (KIF3A and IFT88), decreased that of WNT5A/β-catenin, and increased that of the adipogenesis markers PPARγ and CEBPα in sWAT. HIFU increased the number of adipocytes in the sWAT and the thickness of sWAT. In conclusion, HIFU could selectively increase sWAT levels by modulating the cilia of ASCs and be used for skin rejuvenation.

## 1. Introduction

Skin aging, the most frequently addressed cosmetic problem, is a complex process with both intrinsic and extrinsic etiologies [1,2,3,4]. Intrinsic aging is a chronological process; however, extrinsic aging is mainly induced by exposure to ultraviolet radiation [1,2,3,4]. Both skin aging processes lead to increased reactive oxygen species levels, which cause DNA damage or increased skin inflammation [5]. Increased oxidative stress induces the upregulation of nuclear factor-κB (NF-κB) [6], which sequentially increases the expression of pro-inflammatory cytokines interleukin (IL)-1, IL-6, and tumor necrosis factor-α (TNF-α) [7]. Moreover, skin aging leads to histological changes, such as decreased thickness of the dermis, especially in the subcutaneous white adipose tissue layer (sWAT) [8,9].

Proliferation and differentiation of precursor cells are required for the renewal of tissue-specific cells for the maintenance of homeostasis and tissue repair in adult tissue [10]. Adipose-derived stem cells (ASCs) are an abundant population of progenitor cells in adipose tissue. ASCs have the potential for multilineage differentiation and can proliferate and differentiate into new adipocytes [11]. The potential for the adipogenic differentiation and proliferation of ASCs is known to decrease with aging [12]. The expression of peroxisome proliferator-activated receptor γ (PPARγ), which is an essential transcriptional signal for regulating adipogenic differentiation and maintaining the adipocyte phenotype, decreases with aging in orbital skin [12].

The primary cilia are antenna-like structures on the cell surface that sense stimuli from the extracellular environment and mediate various cell signal transduction pathways, such as Hedgehog signaling [13,14] and canonical and non-canonical Wnt signaling [15].

Since change in cilia length by assembly or disassembly is coupled with control of the cell cycle, primary cilia are involved in cell growth and proliferation [13,16]. Moreover, primary cilia are essential for maintaining the stemness (the ability for self-renewal and differentiation) of stem cells or progenitor cells [17,18,19]. In fact, the length of the primary cilium changes during the ASC differentiation process [17,19]. During adipogenic differentiation of mesenchymal stem cells (MSCs), the number of ciliated MSCs is increased [20]. Deletion of intraflagellar transport protein 88 (IFT88), an essential cilia protein, leads to the destruction of primary cilia formation and reduced adipogenesis [20]. Wnt5a/β-catenin signaling, which inhibits adipogenic differentiation [21], is decreased by the ciliation of MSCs during adipogenesis [20]. It has been suggested that primary cilia are involved in the inhibition of the Wnt signaling pathway, since the deletion of cilia protein, kinesin superfamily protein 3 (KIF3), leads to the destruction of cilia and upregulation of Wnt signaling [22]. It is also known that Wnt/β-catenin signaling leads to downregulation of CCAAT/enhancer binding protein α (C/EBPα) as well as PPARγ, which are main master regulators of adipogenesis [23].

It has been reported that IL-6- and TNF-α-treated trophoblastic cells show decreased primary cilia length [24]. Furthermore, ASCs obtained from obese patients showed de-creased cilia length, which was associated with increased inflammatory cytokines in adipose tissue [25,26,27].

Levels of heat shock proteins (HSP) are enhanced by environmental or pathophysiological stresses, such as mechanical stress, heat shock, fever, inflammation, and infection. HSP levels are also increased by stress during the normal developmental process [28,29,30]. It is reported that HSP70 increases stem cell survival [31]. HSP70 also leads to the downregulation of NF-κB activation, which is induced by lipopolysaccharide (LPS) exposure [32]. HSP70 led to the suppression of inflammatory cytokines, such as IL-6 and TNF-α, in LPS-activated monocytes [33]. Modulation of NF-κB by HSP70 has usually been reported in immune cells, such as macrophages [32]. However, HSP also decreased TNF-α by decreasing NF-κB activation in respiratory epithelial cells [34].

High-intensity focused ultrasound (HIFU) devices generate energy that induces rapid heating of adipose tissue; thus, HIFU causes heat injury and disruption to adipocytes, which induces adipocyte necrosis [35,36,37]. HIFU has also been used for skin rejuvenation by enhancing collagen synthesis [35]. Necrotic debris from adipocytes stimulates the activation of macrophages, which leads to the upregulation of collagen synthesis by fibroblasts [35]. Moreover, HIFU leads to the upregulation of HSPs, such as HSP27, HSP60, HSP70, HSP72, and HSP73 [38,39,40,41,42,43].

Although it is well known that HIFU induces collagen synthesis and increases the expression of HSPs, it has not been fully revealed whether HIFU promotes adipogenesis by stimulating skin ASCs. This study hypothesized that HIFU induces increase in HSP70, which, in turn, leads to decreased activation of NF-κB, IL-6, and TNF-α in sWAT. The decrease in these inflammatory cytokines leads to the restoration of the primary cilia function of ASCs in sWAT. Restored cilia function can decrease Wnt5/β-catenin, which eventually leads to increased PPARγ and CEBPα expression. Increased PPARγ and CEBPα levels lead to increased adipogenesis in subcutaneous adipocytes, which eventually results in increased sWAT thickness. Finally, increased sWAT thickness contributes to facial skin rejuvenation. Thus, we evaluated the effect of HIFU on subcutaneous adipogenesis via modulation of the cilia of ASCs in rat skin.

## 2. Results

### 2.1. HIFU Increased Levels of HSP70 and Decreased Those of NF-κB, IL-6, and TNF-α

First, it was evaluated whether HSP70 expression was increased in sWAT after HIFU application. HIFU was applied to rat skin at 0.2 J and the skin was harvested at 1, 3, 7, and 28 days after HIFU application. When HIFU was applied at 0.2 J to swine skin, the tissue was heated to approximately 40 °C (Table S1).

**Figure 1:**
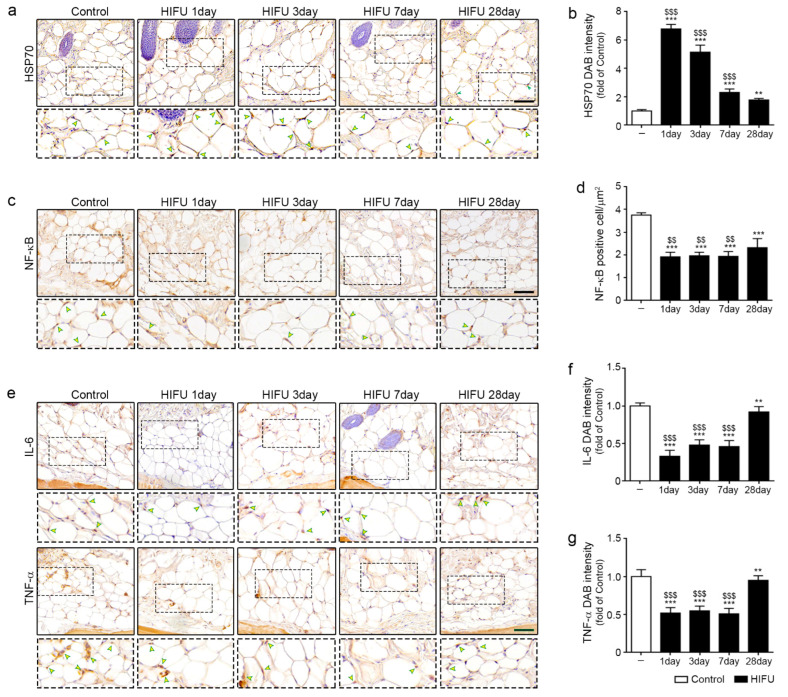
Attenuated effect of NF-κB/IL-6/TNF-α by increase in HSP70 in sWAT due to HIFU. (**a**) HSP70 expression (arrows) in the sWAT of rats was confirmed by immunohistochemistry (scale bar = 100 μm). (**b**) Quantification of the HSP70 DAB staining image in (**a**). The intensity of HSP70 was highest 1 day after HIFU application and gradually decreased over time. (**c**) NF-κB positive cells (arrows) in the sWAT of rats were confirmed by immunohistochemistry (scale bar = 100 μm). (**d**) Quantification of NF-κB-positive cells per μm^2^ in (**c**). The number of NF-κB positive cells in the sWAT at 1, 3, 7, and 28 days after HIFU application was significantly lower than that of the control. (**e**) IL-6 and TNF-α expression (arrows) in the sWAT of rats were confirmed by immunohistochemistry (scale bar = 100 μm). (**f**) Quantification of the IL-6 DAB staining image in the upper row of (**e**). The expression of IL-6 was lowest 1 day after HIFU application, and it increased over time. (**g**) Quantification of the TNF-α DAB staining image in the lower row of (**e**). The expression of TNF-α in the sWAT at 1, 3, 7, and 28 days after HIFU application was significantly lower than that in the control. All intensities were analyzed against the level of the control group. Three animals were used in each group and data are represented as the means ± SDs. **, *p* < 0.01 and ***, *p* < 0.001, vs. Control; $$, *p* < 0.01 and $$$, *p* < 0.001, vs. HIFU 28 day. DAB, 3,3-Diaminobenzidine; HIFU, high-intensity focused ultrasound; HSP, heat shock protein; IL-6, interleukin-6; NF-κB, nuclear factor-κB; SD, standard deviation; sWAT, subcutaneous adipose tissue; TNF-α, tumor necrosis factor-alpha.

Expression of HSP70 was determined using HSP70 3,3-diaminobenzidine (DAB) staining. The expression of HSP70 in sWAT was significantly higher 1 day after HIFU application than in the control, which was not applied with HIFU. The expression of HSP70 was highest 1 day after HIFU application and gradually decreased over time (Figure 1a,b).

The expression of NF-κB in sWAT at 1, 3, 7, and 28 days after HIFU application was significantly lower than that in the control (Figure 1c,d). The expression of IL-6 in sWAT was decreased by HIFU compared to that in the control. The expression of IL-6 was lowest 1 day after HIFU application. It increased with time; however, the expression of IL-6 at 28 days after HIFU application was still lower than that of the control (upper rows of Figure 1e,f). The expression of TNF-α in sWAT at 1, 3, 7, and 28 days after HIFU application was significantly lower than that in the control (lower rows of Figure 1e,g).

### 2.2. HIFU Decreased Expression of Cilia Disassembly Protein in the ASCs

Aurora kinase A (AurA) and histone deacetylase 6 (HDAC6) are the main players involved in cilia disassembly [44]. We performed co-staining of AurA or HDAC6 with CD166, an ASC marker [45], to evaluate cilia disassembly in the ASCs of sWAT.

**Figure 2:**
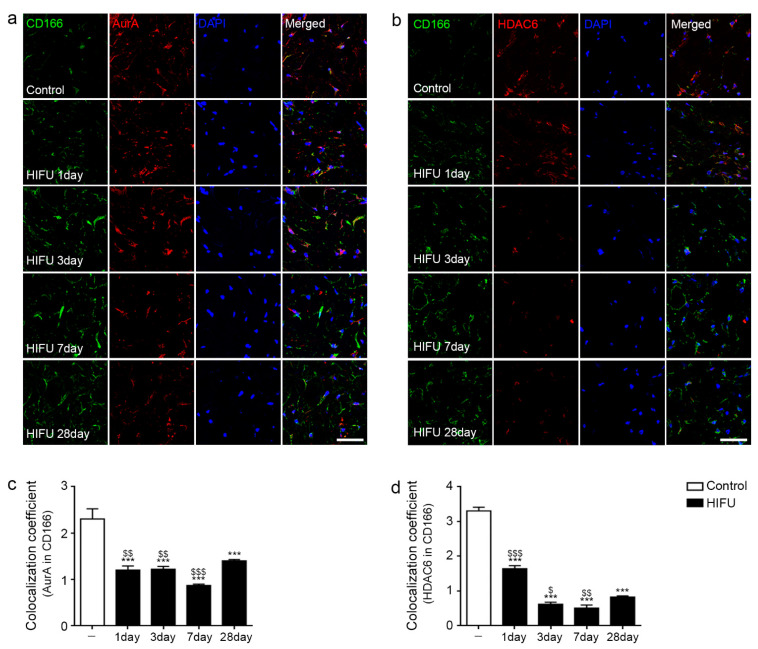
Decrease in cilia disassembly protein of ASCs in sWAT after HIFU application. (**a**) CD166 (ASC marker; green), AurA (red), and DAPI (nuclei marker; blue) in the sWAT of rats were identified by immunofluorescence (scale bar = 50 μm). (**b**) CD166 (ASC marker; green), HDAC6 (red), and DAPI (nuclei marker; blue) in the sWAT of rats were confirmed by immunofluorescence (scale bar = 50 μm). (**c**) Colocalization coefficient of AurA in CD166 using the image in (**a**). The expression of AurA in the ASCs was significantly decreased by HIFU compared to that in the control. (**d**) Colocalization coefficient of HDAC6 in CD166 using the image in (**b**). The intensity of HDAC6 in the ASCs was decreased compared to that in the control from 1 day after HIFU application and it decreased more at 3 and 7 days after HIFU application. All intensities were analyzed against the level of the control group. Three animals were used in each group and data are represented as the means ± SDs. ***, *p* < 0.001, vs. Control; $, *p* < 0.05, $$, *p* < 0.01 and $$$, *p* < 0.001, vs. HIFU 28 day. ASCs, adipose-stem cells; AurA, aurora kinase A; CD166, cluster of differentiation 166; DAPI, 4′,6-diamidino-2-phenylindole; HDAC6, histone deacetylase 6; HIFU, high-intensity focused ultrasound; SD, standard deviation; sWAT, subcutaneous adipose tissue.

The expression of AurA in ASCs was significantly decreased by HIFU compared with that in the control. The expression of AurA in ASCs was lowest 7 days after HIFU application (Figure 2a,c).

The expression of HDAC6 in ASC was decreased compared with that in the control from 1 day after HIFU application, and the decrease was greater at 3 and 7 days after HIFU application (Figure 2b,d).

### 2.3. HIFU Increased Levels of Cilia Assembly Proteins and Decreased Those of Wnt5a/β-Catenin

**Figure 3:**
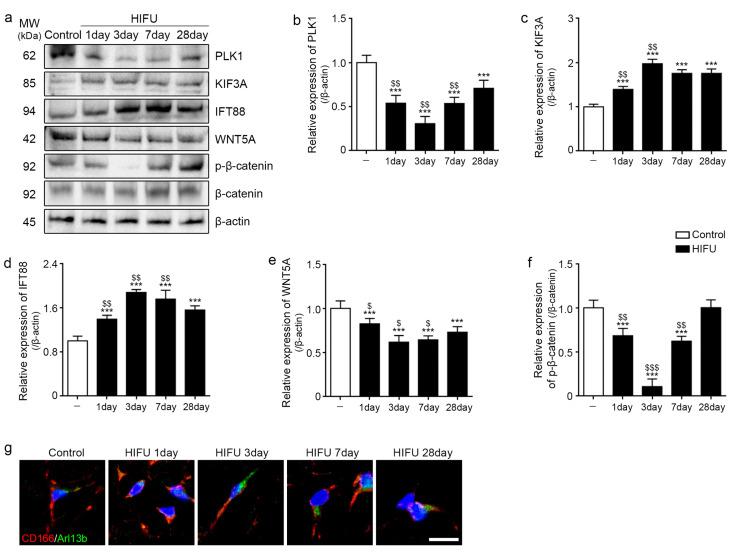
Increased cilia assembly protein and decreased WNT5A/β-catenin in sWAT after HIFU application. (**a**) All protein levels in the sWAT of rats were measured using Western blotting. (**b**) The protein expression of PLK1 was decreased from 1 day after HIFU application compared to that in the control. (**c**,**d**) The protein expression of KIF3A and IFT88 in sWAT was significantly increased after HIFU application compared to that in the control. (**e**,**f**) The protein expression of WNT5A and p-β-catenin/β-catenin in sWAT was decreased after HIFU application compared to that in the control. (**g**) CD166 (ASC marker; red), Arl13b (cilia-specific marker; green), and DAPI (nuclei marker; blue) in the sWAT of rats were identified by immunofluorescence (scale bar = 10 μm). Three animals were used in each group and data are represented as the means ± SDs. ***, *p* < 0.001, vs. Control; $, *p* < 0.05, $$, *p* < 0.01 and $$$, *p* < 0.001, vs. HIFU 28 day. Actb, actin beta; β-catenin, beta-catenin; HIFU, high-intensity focused ultrasound; IFT88, intraflagellar transport protein 88; KIF3a, kinesin family member 3A; PLK1, polo-like kinase 1; SD, standard deviation; sWAT, subcutaneous adipose tissue; WNT5A, Wnt family member 5a.

Polo-like kinase 1 (PLK1) is also involved in ciliary disassembly [13]. The expression of PLK1 was decreased 1 day after HIFU application compared to that in the control. It was lowest three days after HIFU application (Figure 3a,b).

The expression of KIF3A and IFT88 was significantly increased from 1 day after HIFU application compared to that in the sWAT of the control, and the expression was highest 3 days after HIFU application (Figure 3a,c,d).

The expression of WNT5A and β-catenin was significantly decreased from 1 day after HIFU application compared to that in the sWAT of the control, and the expression was lowest 3 days after HIFU application (Figure 3a,e,f).

To evaluate cilia morphology change in ASCs, co-staining of cilia-specific marker, Arl13b (green) and the ASC marker (CD166; red) was performed. The cilia were most elongated at 3 days after HIFU application (Figure 3g).

### 2.4. HIFU Induced Adipogenesis in sWAT

**Figure 4:**
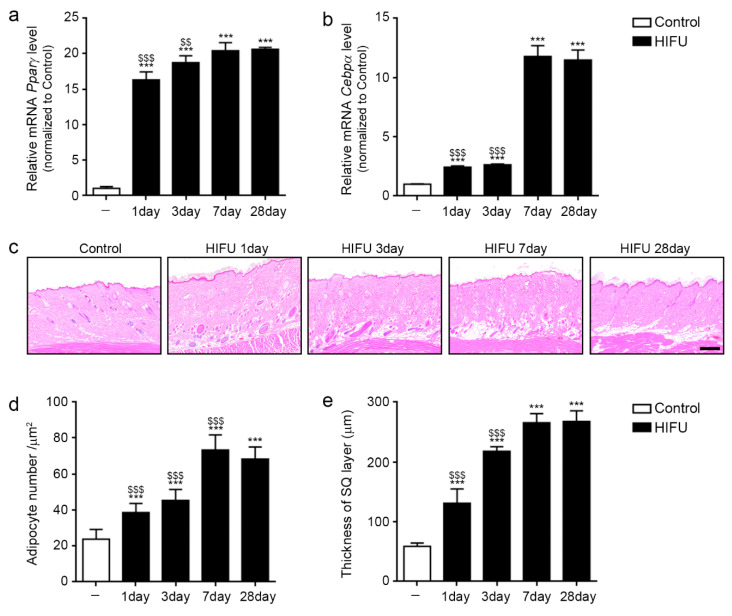
Induction of adipogenesis in sWAT after HIFU application. All mRNA levels in the sWAT of rats were measured using qRT–PCR. (**a**,**b**) The mRNA expression of Pparγ and Cebpα was increased from 1 day after HIFU application compared to that in the control. (**c**) The adipocyte number and thickness of sWAT were confirmed by H&E staining (scale bar = 100 μm). (**d**,**e**) Quantification of the H&E staining image in Figure 4c. The number of adipocytes in sWAT at 1 day after HIFU was significantly increased compared to that in the control (**d**), and the thickness of sWAT was significantly increased by HIFU and increased with time (**e**). Three animals were used in each group and data are represented as the means ± SDs. ***, *p* < 0.001, vs. Control; $$, *p* < 0.01 and $$$, *p* < 0.001, vs. HIFU 28 day. Cebpα, CCAAT/enhancer-binding protein alpha; H&E, hematoxylin and eosin; HIFU, high-intensity focused ultrasound; Pparγ, peroxisome proliferator-activated receptor-gamma; qRT-PCR, quantitative reverse transcription polymerase chain reaction; SD, standard deviation; sWAT, subcutaneous adipose tissue.

The mRNA expression of the adipogenesis markers Pparγ and Cebpα in sWAT 1 day after HIFU application was significantly increased compared to that in the control. These expression levels were the highest 7 days after HIFU application (Figure 4a,b).

The number of adipocytes in sWAT 1 day after HIFU application was significantly higher than in the control, and it was highest 7 days after HIFU application (Figure 4c,d).

The thickness of sWAT was significantly increased by HIFU and increased over time (Figure 4c,e).

## 3. Discussion

Recently, sWAT has been considered a target for facial skin rejuvenation due to facial sWAT changing during the skin aging process [46,47,48,49]. Both the structure and volume of sWAT undergo various changes during aging [50,51]. Adipogenesis potential also decreases with age, which is accompanied by decreased expression of PPRAγ [52]. These changes induce alterations in the mechanical properties of skin and lead to decreased critical mechanical strain, which is required for structural stability, eventually generating wrinkles in aged skin [53].

Primary cilia length is controlled through the processes of assembly and disassembly and is involved in cell cycle control [54,55]. When cilia are formed, the cell exits the cell cycle. In contrast, the cilia are disassembled when the cell enters the cell cycle [54,55]. Moreover, primary cilia are involved in the maintenance and differentiation of various stem cells, including MSCs [18]. Cilia loss induced by knockdown of IFT88 or KIF3A causes defects in adipogenic differentiation, which is accompanied by decreased expression of PPARγ in MSCs [20]. Cilia loss induced by KIF3A deletion in mouse embryonic stem cells and mouse embryonic fibroblasts enhanced Wnt/β-catenin signaling [22]. Based on these factors, it was hypothesized that cilia length control in ASCs could lead to adipogenesis in sWAT and promote skin rejuvenation.

HIFU devices have been used in noninvasive subcutaneous lipolysis for ablating unwanted adipose tissue. HIFU induces immediate destruction of cell membranes [36]. Moreover, heat generated by HIFU damages extra adipocytes at temperatures of 58 °C, which results in coagulative necrosis [36]. For body sculpting (subcutaneous lipolysis), HIFU is usually applied at a total energy of 140 J/cm^2^ or higher [35]. HIFU is also used to enhance collagen synthesis and decrease skin laxity [56]. For decreasing skin laxity, HIFU is usually applied at 10 MHz (0.1–0.25 J), with a focal depth of 3 mm and a frequency of 7 MHz (0.1–1 J), or with a focal depth of 4.5 mm and a frequency of 4 MHz (0.1–1.25 J), depending on skin thickness [56].

Increased levels of pro-inflammatory cytokines, such as IL-6 and TNF-α, result in decrease in cilia length [24]. HSP70 is known to decrease inflammatory cytokines, such as IL-6 and TNF-α [32].

A previous study showed that heat shock, which was induced by mild hyperthermia around 43 °C, led to the upregulation of HSP27, HSP70, and HSP90 in dental pulp and dental pulp cells [57]. Moreover, several studies have shown that heat shock can be an effective way to increase cell survival during cell culture [58,59]. Heat shock-pretreated MSCs (42 °C for 1 h) showed increased transplanted cell survival in liver ischemia–reperfusion injury [60,61]. Heat shock pretreatment at 42 °C promoted the upregulation of HSP70 in MSCs and decreased activation of NLR family pyrin domain containing 3 inflammasome in alveolar macrophages [62]. Moreover, the upregulation of HSP70 in MSCs by preheating treatment decreased levels of pro-inflammatory cytokines, such as IL-6 and TNF-α, which eventually decreased acute lung injury [62].

In this study, we aimed to restore the cilia function of ASCs, which was decreased by pro-inflammatory cytokines via the upregulation of HSP70. Since HIFU was used for increasing HSP70 expression and not for adipocyte ablation, the target temperature generated by HIFU was maintained at approximately 40 °C. To achieve this temperature, HIFU was applied at 0.2 J. The results showed that expression of HSP70 in sWAT was increased by HIFU at 0.2 J. Expression of HSP70 expression was highest 1 day after HIFU and decreased over time. However, HSP70 expression was higher than that in the control group until 28 days after HIFU application. In contrast, HIFU decreased the expression of NF-κB, IL-6, and TNF-α in sWAT. Next, we evaluated whether decrease in levels of pro-inflammatory cytokines was associated with the restoration of cilia function in ASCs. ASCs in sWAT showed decreased expression of cilia disassembly proteins, including AurA and HDAC6, following HIFU treatment. The expression of PLK1, which is involved in cilia disassembly, was also decreased by HIFU in sWAT. In contrast, the expression of KIF3a and IFT88, which are involved in cilia assembly, was increased by HIFU in the sWAT. The expression of Wnt5a/β-catenin, which is known to be negatively associated with cilia length, was decreased by HIFU in sWAT. Furthermore, the expression of PPARγ and CEBPα, which are markers of adipogenesis, was increased by HIFU. As adipogenesis signals increased, the adipocyte number in and the thickness of sWAT were increased by HIFU.

It seemed that HIFU could increase adipogenesis in sWAT by increasing HSP70 and decreasing pro-inflammatory cytokines. One possible mechanism which could have been responsible for the increased adipogenesis in sWAT might have been an attenuation effect of HSP70 that decreased inflammation in the adipose tissue, which modulated the cilia length of ASCs. We cannot say that increased HSP70 by HIFU was directly involved in decreasing pro-inflammatory cytokines, since we did not use HSP70 knockout models. In a future study, the exact mechanism of HIFU-induced ASC cilia modulation should be evaluated with HSP70 knockout models.

Although interest in sWAT as a new target for facial rejuvenation has increased, there has been no specific method reported that can selectively increase sWAT thickness or volume, especially in the face. Thus, it was hypothesized that HIFU could be a method to increase sWAT volume by increasing the expression of HSP70, which is upregulated by hyperthermia. HIFU (0.2 J) effectively upregulated HSP70 expression. HIFU has been used for adipose tissue ablation or collagen denaturation, with the target temperature generated in the tissue being over 58 °C [36]. Our study showed that HIFU at 0.2 J could upregulate HSP70, which modulated adipogenesis without adipocyte ablation. Those results suggests that HIFU could be a possible application for facial rejuvenation, even though future clinical studies should be performed to obtain conclusive evidence.

**Figure 5:**
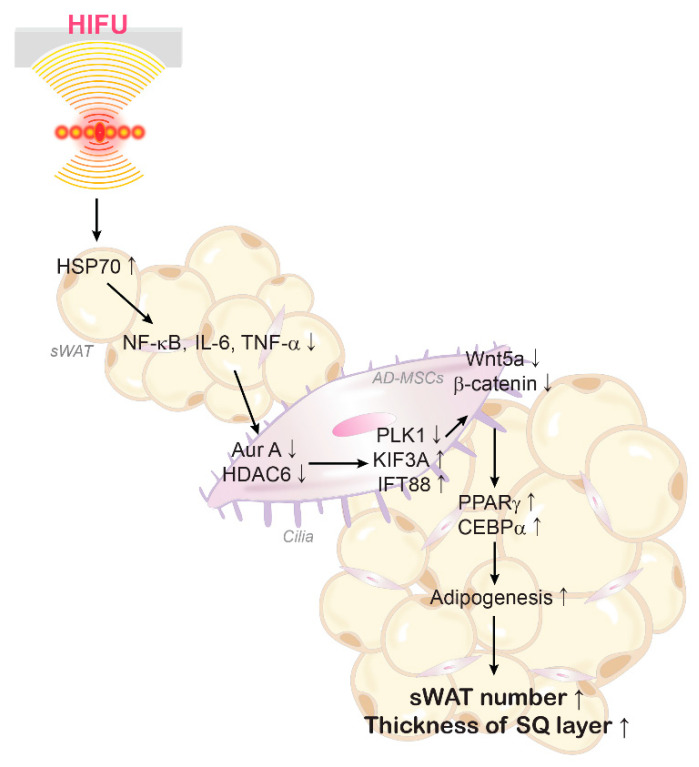
Summary of this study.

In conclusion, HIFU induced upregulation of HSP70, which decreased the expression of NF-κB, IL-6, and TNF-α in sWAT. Decrease in levels of inflammatory cytokines was accompanied by decrease in expression of cilia disassembly proteins and increase in expression of assembly proteins and Wnt5a/β-catenin. The expression of adipogenesis signals (PPARγ and CEBPα), number of adipocytes, and the thickness of sWAT were increased by HIFU in the animal skin (Figure 5).

## 4. Materials and Methods

### 4.1. HIFU System

A HIFU system (LinearZ, Jeisys Medical Inc., Seoul, Korea) was used in this study. The HIFU system utilizes the thermal action of the energy output from a focused ultrasound transducer. The transducer has its natural frequency and operates at 2 MHz (focal depth 9.0–13.0 mm), 4 MHz (focal depth 4.5–6.0 mm), or 7 MHz (focal depth 1.0–3.0 mm). The system has two modes: DOT and LINEAR. The DOT mode forms focal points at a certain distance (adjustment of 1.0–2.0 mm), whereas the LINEAR mode creates linear energy without any space between the focusing points. The maximum output energy of the system was 3.0 J.

This study used the 7 MHz transducer and applied DOT modes at 7 MHz at 2.0 mm focal depths. The applied energy was 0.2 J.

### 4.2. Method of Temperature Measurement

Porcine fat was selected as the sample for temperature measurement. The fat sample was prepared with a thickness of >6 cm, and a thermometer (Fluoroptic^®^ Thermometer m3300 Biomedical Lab Kit, Luxtron Corp, Santa Clara, CA, USA) was inserted at the transducer’s focal depth.

The test sample was immersed in a hot water tank to heat it to 35–37 °C (approximately human body temperature). Ultrasound gel was applied to the sample surface, and the cartridge contacted the sample surface. The HIFU energy was irradiated in one shot, and the peak temperature of the focused energy was measured. The measurements were performed three times under the same conditions and the average value was calculated (Table S1).

### 4.3. Animal Experiments and HIFU Application

All animal experiments were conducted in accordance with the guidelines of the Institutional Animal Care and Use Committee and approved by the Center of Animal Care and Use Ethical Board of Gachon University (approval number: LCDI-2021-0135).

Eight-week-old male Sprague-Dawley rats (220 ± 20 g) were obtained from Orient Bio (Sungnam, Korea). The rats were housed in cages with a 12 h light/dark cycle under a controlled temperature (22 ± 5 °C) and relative humidity (50 ± 10%) and had free access to a standard laboratory diet and water.

After acclimatization for 1 week, the rats were randomly divided into five groups, three rats per group, as follows: HIFU 1 day, HIFU 3 day, HIFU 7 day, and HIFU 28 day, from which samples were collected 1, 3, 7, and 28 days after HIFU application, respectively, and a control group (no HIFU application). The mice were subjected to HIFU (DOT mode, depth 2 mm, at 0.2 J), and the skin and sWAT were dissected from euthanized rats after 1, 3, 7, and 28 days of HIFU application. Skin and sWAT tissues were collected immediately for paraffin block embedding, and a sample for protein isolation was collected by separating the skin and sWAT.

### 4.4. Preparation of Paraffin-Embedded Skin and sWAT Tissue Sections

The skin tissues attached to sWAT were fixed with 4% paraformaldehyde (Sigma-Aldrich, St. Louis, MO, USA). Fixed tissue samples were washed for 30 min for embedding. Paraffin blocks of skin tissues attached to sWAT were then made using a tissue processor (Thermo Fisher Scientific, Waltham, MA, USA). The paraffin-embedded blocks were sectioned at 7 µm using a microtome (Leica, Wetzlar, Germany) and dried at 37 °C for 24 h to keep them attached to the coated slides. The sectioned slides were reacted with xylene and descending ethanol series (100%, 95%, 80%, and 70%) for deparaffination.

### 4.5. DAB Staining

The deparaffinized slides of skin tissues attached to sWAT were loaded with 3% hydrogen peroxide–methanol for 30 min at room temperature to block endogenous peroxidase. After washing with phosphate-buffered saline (PBS), the slides were boiled in an antigen retrieval solution (sodium citrate buffer, pH 6.0) using a microwave. The tissue slides were rinsed with PBS and blocked with normal animal serum for 1 h at room temperature. The slides were then incubated with primary antibodies (Table S2) in normal serum for 24 h at 4 °C. The slides were rinsed with PBS, tagged with a biotinylated antibody in normal animal serum (1:200 dilution; Vector Laboratories Inc., Burlingame, CA, USA) for 2 h and then incubated with the ABC reagent (Vector Laboratories Inc.) for 30 min at room temperature. The tagged slides were washed with PBS, developed using 3,3′-diaminobenzidine (Sigma-Aldrich) for 15 min to identify the brown signal, and washed with running water for 10 min.

To confirm the nuclei, tissue slides were stained with hematoxylin solution (Korea Pathology Technical Center; KPNT, Cheongju, Korea) for 1 min and then placed in ascending ethanol series (from 70% to 100%) for 5 min. Finally, the slides were mounted with dibutylphthalate polystyrene xylene mounting solution (DPX; Sigma-Aldrich).

Images of the stained tissues were captured under an optical microscope (Olympus Optical Co., Tokyo, Japan) and analyzed using ImageJ software (NIH, Bethesda, MD, USA).

### 4.6. Immunofluorescence

As described in Section 4.5, normal animal serum was loaded onto the antigen-recovered slides to block nonspecific sites. The tissue slides were then incubated with primary antibodies (Table S2) overnight at 4 °C. The slides were then washed with PBS and incubated for 1 h at room temperature with the appropriate secondary antibodies conjugated to AlexaFluor-488 and AlexaFluor-555 in normal animal serum (1:500 dilution; Invitrogen, Waltham, MA, USA). The conjugated tissue slides were washed again in PBS and stained with 4′, 6-diamidino-2-phenylindole (DAPI; Sigma-Aldrich) for 5 min at room temperature to identify the nuclei. The stained slides were mounted using vector shield solution (Vector Laboratories, CA, USA) for confocal imaging. The prepared slides were visualized using a confocal microscope (LSM 710, Carl Zeiss, Oberkochen, Germany) at the Core Facility for cell-to-in vivo imaging. At least ten confocal images were randomly captured for image analysis and merged fluorescence intensity was analyzed using ZEN 2009 software (Carl Zeiss).

### 4.7. Western Blots

Frozen sWAT tissues were homogenized by Bioprep-24R (Allsheng, Hangzhou, China) using an EzRIPA lysis kit (ATTO, Tokyo, Japan) according to the manufacturer’s instructions.

Thirty micrograms of protein were loaded into 4–12% polyacrylamide gels for separation by electrophoresis (Criterion System, Bio-Rad Laboratories, Inc., Hercules, CA, USA). Proteins were transferred onto PVDF membranes (Merck Millipore, Burlington, MA, USA) and incubated overnight with the primary antibodies listed in Table S3. Proteins were visualized using horseradish peroxidase-conjugated secondary antibodies (Vector Laboratories Inc.) and enhanced chemiluminescence (ECL) substrate (Cytiva, Vancouver, BC, Canada) on a digital acquisition system (Bio-Rad). Individual protein expression values were quantified using Image J software (NIH) [63] and normalized to beta-actin to control for differences in protein loading. Values for a single blot were expressed relative to the mean of the control group.

### 4.8. Hematoxylin and Eosin Staining

To measure the number of adipocytes and the thickness of sWAT, the skin tissues attached to sWAT were stained with hematoxylin and eosin. Briefly, deparaffinized and rehydrated tissue slides were immersed in hematoxylin solution (KPNT) and washed with tap water for 3 min. Tissue slides were then immersed in eosin solution (KPNT) for 1 min and rinsed with running water. The cover slips were mounted using DPX solution (Sigma-Aldrich), and slides were visualized under an optical microscope (BX53M; Olympus, Japan). The adipocyte number in and thickness of the sWAT were determined by randomly capturing 10 sWAT images using Image J software (NIH).

### 4.9. Statistical Analysis

Data were validated from at least three replicates for each experiment and are presented as means ± standard deviations. In this study, the Kruskal–Wallis test was used for comparisons of five groups and the Mann–Whitney U test for a post hoc analysis using SPSS v.22 (IBM Corporation; Armonk, NY, USA). Statistical significance was represented as follows: *, vs. Control; $, HIFU 28 day.

## Data Availability

All data are contained within the article.

## Supplementary Materials

The following supporting information can be downloaded at: https://www.mdpi.com/article/10.3390/ijms23168866/s1.
