# Supplementary material for: High-Intensity Focused Ultrasound Induces Adipogenesis via Control of Cilia in Adipose-Derived Stem Cells in Subcutaneous Adipose Tissue
Source: Int J Mol Sci. 2022 Aug 9;23(16):8866. doi: 10.3390/ijms23168866 (PMC9408610; doi:10.3390/ijms23168866)
Supplement: Supplementary file 1 [file ijms-23-08866-s001.zip › ijms-1836606-supplementary.pdf]

International Journal of
*Molecular Sciences*

# Supplementary Tables

**Table S1. Temperature according to HIFU applied energy**

| J | °C |
|---|---|
| 0.2 | 40 |
| 0.3 | 42.7 |
| 0.4 | 47.2 |
| 0.5 | 49.4 |
| 0.6 | 54.1 |
| 0.7 | 58.1 |
| 0.8 | 60.7 |
| 0.9 | 63.5 |
| 1 | 66.3 |

**Table S2. List of antibodies used for immunohistochemistry and immunofluorescence**

| Antigen (host) | Company | Dilution rate | |
|---|---|---|---|
| | | Immunohistochemistry | Immunofluorescence |
| HSP70 (rabbit) | Cell Signaling Technology | 1 : 200 | |
| NF-κB (rabbit) | Cell Signaling Technology | 1 : 400 | |
| IL-6 (rabbit) | Abcam | 1 : 100 | |
| TNF-α (rabbit) | Novus biologicals | 1 : 200 | |
| CD166 (mouse) | HUABIO | | 1 : 50 |
| AurA (rabbit) | Fine Test | | 1 : 100 |
| HDAC6 (rabbit) | Abclonal | | 1 : 100 |
| Arl13b (rabbit) | Proteintech | | 1 : 50 |

**Table S3. List of antibodies used for western blot**

| Antigen (host) | Company | Dilution rate |
|---|---|---|
| PLK1 (rabbit) | Cell Signaling Technology | 1 : 1,000 |
| KIF3a (rabbit) | Affinity | 1 : 1,000 |
| IFT88 (rabbit) | Affinity | 1 : 1,000 |
| WNT5A (rabbit) | Invitrogen | 1 : 1,000 |
| β-catenin (mouse) | Santa cruz biotechnology | 1 : 300 |
| p-β-catenin (rabbit) | Cell Signaling Technology | 1 : 1,000 |
| β-actin (rabbit) | Cell Signaling Technology | 1 : 1,000 |
